# Simultaneous Trace Analysis of Lead and Cadmium in Drinking Water, Milk, and Honey Samples Through Modified Screen-Printed Electrode

**DOI:** 10.3390/bios15050267

**Published:** 2025-04-23

**Authors:** Fei Wang, Xiao Peng, Ziqian Xiao, Ying Ge, Bilin Tao, Zhaoyong Shou, Yifei Feng, Jing Yuan, Liang Xiao

**Affiliations:** 1 Faculty of Naval Medicine, Naval Medical University, No. 800 Xiangyin Road, Yangpu District, Shanghai 200433, China; feiwang116@163.com (F.W.); xiaopeng57827@163.com (X.P.); xiaoziqian130@163.com (Z.X.); nmuyingge@foxmail.com (Y.G.); bltao@smmu.edu.cn (B.T.); 2 Faculty of Health Service, Nacal Medical University, Shanghai 200433, China; szy@smmu.edu.cn; 3 Naval Special Medical Center, Naval Medical University, Shanghai 200433, China; fengyifei1012@163.com; 4 Department of Pediatrics, Changhai Hospital, Naval Medical University, No. 168 Changhai Road, Yangpu District, Shanghai 200433, China

**Keywords:** N-doped reduced graphene oxide coated with polypyrrole, simultaneous detection of heavy metal ions, electrochemical sensor, multiple-sample testing

## Abstract

A composite (N-rGO@ppy) of N-doped reduced graphene oxide (N-rGO) coated with polypyrrole (ppy) particles was successfully synthesized. The incorporation of N-rGO significantly mitigates the aggregation of ppy synthesized in situ, and the doped N atoms improve the conductivity of graphene oxide (GO), thereby enhancing N-rGO@ppy’s redox properties. Firstly, a glassy carbon electrode (GCE) modified with N-rGO@ppy (N-rGO@ppy/GCE) was used in combination with a bismuth film and square-wave anodic stripping voltammetry (SWASV) for the simultaneous trace analysis of Pb^2+^ and Cd^2+^. N-rGO@ppy/GCE exhibited distinct stripping peaks for Pb^2+^ and Cd^2+^, with a linear range of 1 to 500 μg L^−1^. The limits of detection (LODs) were found to be 0.080 μg L^−1^ for Pb^2+^ and 0.029 μg L^−1^ for Cd^2+^, both of which are significantly below the standards set by the World Health Organization (WHO). Subsequently, the same electrochemical sensing strategy was adapted to a more portable screen-printed electrode (SPE) to accommodate the demand for in situ detection. The performance of N-rGO@ppy/SPE for analyzing Pb^2+^ and Cd^2+^ in actual samples, such as drinking water, milk, and honey, showed results consistent with those obtained from conventional graphite furnace atomic absorption spectrometry (GFAAS).

## 1. Introduction

Heavy metals have emerged as a significant source of environmental pollution, infiltrating air, soil, water, and food due to increasing anthropogenic industrial and agricultural activities [1]. Among them, cadmium and lead, as prominent examples of heavy metals, have been linked to diseases affecting the human cardiovascular, renal, and nervous systems. Additionally, their ability to accumulate in the food chain poses toxicity risks even at low levels [2]. Therefore, sensitive and rapid monitoring of cadmium and lead levels in drinking water and food samples is essential.

Water plays a crucial role in the human body’s metabolic processes, and the WHO recommends maximum permissible limits of 10 μg L^−1^ for Pb^2+^ and 3 μg L^−1^ for Cd^2+^ in drinking water [3]. Furthermore, international and national regulatory agencies are also working to lower acceptable heavy metal levels in food products [4]. Numerous methods have been developed for the determination of heavy metals, including traditional techniques such as atomic absorption spectrometry (AAS) [5], atomic fluorescence spectrometry (AFS) [6], and inductively coupled plasma mass spectrometry (ICP-MS) [7], as well as portable near-infrared spectroscopy (p-NIR) [8] for on-site detection. While these methods can accurately measure the concentrations of Pb^2+^ and Cd^2+^, they come with limitations, including complicated sample preparation, long detection times, expensive equipment, and the need for skilled personnel [9]. In contrast, electrochemical techniques, such as potentiometry [10] and square-wave voltammetry (SWV), offer several advantages, including flexibility, simplicity, speed, and high sensitivity [11]. Among these techniques, SWASV is widely used for heavy metal detection and involves two main processes: the preconcentration of analytes and electrochemical stripping [12].

Researchers have conducted extensive studies on electrode materials to enhance the redox reactions of heavy metal ions at the surface of the working electrode (WE) [13,14,15]. The bismuth-film electrode has emerged as a promising alternative to the mercury film electrode, offering excellent electrochemical properties and environmental benefits [16]. Moreover, other materials, such as gold nanoparticles, conductive polymers, biochemical substances, and carbon-based materials, have also been applied [17]. Among them, polymers, especially ppy, are one of the most significant conductive polymers, and they serve to adsorb and detect heavy metal ions. However, ppy invariably suffers from slight solubility in water and severe aggregation, further causing poor dispersion and machinability. The incorporation of nanomaterials such as GO and carbon nanotubes (CNTs) can further improve the solubility and electrocatalytic properties of ppy [18,19]. GO possesses unique properties, such as a high surface area, significant mechanical strength, high π-conjugation, good chemical stability, and hydrophilic properties. Beyond that, various functional groups of the GO surface can serve as active sites for further modification. Although GO has many advantages for electrochemical detection, the unsolved issues are still the redox properties and the poor conductivity [20,21,22]. In order to solve these problems, N atoms were introduced to GO. N has one more valence electron than C, resulting in new energy levels, and the electrical properties of N-rGO were changed by the new energy levels, lowering part of the conduction band for sp^2^-bonded C nanostructures such as reduced graphene oxide (rGO), which can further improve the redox properties and conductivity [19,20]. Taking into account these aspects, our goal was to design a scheme for obtaining a novel material by polymerizing the pyrrole monomer along the surface of N-rGO, allowing for detecting heavy metal ions.

In our study, urea was incorporated into the GO matrix as a N source to enhance conductivity. By grafting ppy molecules onto the N-rGO surface, the problem of ppy aggregation was effectively solved, which improved the heavy metal ion enrichment ability and detection sensitivity. The synthesized N-rGO@ppy composite material, which features amino and other functional groups serving as active sites, demonstrates an enhanced ability to capture heavy metal ions. As expected, N-rGO@ppy/GCE, in combination with a bismuth film utilized for SWASV in this paper, exhibits remarkable electrochemical properties, with Pb^2+^ and Cd^2+^ determined as having LODs of 0.080 μg L^−1^ and 0.029 μg L^−1^, respectively. Based on the electrochemical sensing strategy validated using GCE, we then modified a more portable commercial SPE with N-rGO@ppy to create N-rGO@ppy/SPE. This modified electrode effectively quantified Pb^2+^ and Cd^2+^ in drinking water, milk, and honey samples, demonstrating high recovery rates. Additionally, the results were consistent with those obtained from traditional GFAAS, highlighting its significant potential for food analysis and environmental monitoring.

## 2. Materials and Methods

### 2.1. Chemicals

All chemicals in this study are of analytical grade and were used directly without further purification. Graphite powder (≥99.99%) was acquired from Sigma-Aldrich. Cd(NO_3_)_2_·4H_2_O, Pb(NO_3_)_2_, pyrrole (Py), and ammonium persulfate (APS) were sourced from Aladdin Biochemical Technology Co., Ltd. (Shanghai, China). K_3_[Fe(CN)_6_] and K_4_[Fe(CN)_6_] were obtained from Sinopharm Chemical Reagent Co., Ltd. (Shanghai, China). Bi(NO_3_)_3_, Nafion (5 wt.%), and urea were purchased from Macklin Biochemical Technology Co., Ltd. (Shanghai, China). The pH standard buffers (pH: 4.00, 6.86, 9.18) were acquired from Shanghai INASE Scientific Instrument Co., Ltd. (Shanghai, China). All other analytical-grade chemicals were sourced from Shanghai Titan Scientific Co., Ltd. (Shanghai, China). A 0.1 M HAc-NaAc buffer solution (pH 4.5) was prepared by mixing appropriate amounts of acetic acid (HAc) and sodium acetate (NaAc) and was employed as the supporting electrolyte. Standard stock solutions of Pb^2+^, Cd^2+^, and Bi^3+^ were prepared from their respective nitrates, Pb(NO_3_)_2_, Cd(NO_3_)_2_·4H_2_O, and Bi(NO_3_)_3_. They were subsequently diluted to the necessary concentrations using the HAc-NaAc buffer solution prior to use. The ultrapure water used for all experiments was supported by Millipore-Q system (Millipore, Bedford, MA, USA).

### 2.2. Apparatus

All electrochemical measurements were performed using an electrochemical workstation (CHI-660E, Chenhua Instrument Co., Ltd., Shanghai, China). A conventional three-electrode system was composed of a bare GCE (d = 4 mm) or modified GCE as the WE, Ag/AgCl as the reference electrode (RE, with saturated KCl solution), and platinum wire as the counter electrode (CE). The commercially available SPE (model: C200) utilized in this study was obtained from Changsha Sunjeen Electronic Technology Co., Ltd. (Changsha, China). Each SPE features a three-electrode configuration consisting of a carbon working electrode (d = 4 mm), a carbon counter electrode, and a Ag/AgCl reference electrode. The overall dimensions of each SPE are 36 × 10 × 0.25 mm. The morphology of the as-prepared materials was observed by scanning electron microscopy (SEM, Hitachi, Tokyo, Japan). X-ray photoelectron spectroscopy (XPS) analysis was conducted on an ESCALAB250 XPS spectrometer with an Mg Kα X-ray source (Boyue Instruments Co., Ltd., Shanghai, China). Fourier transform infrared spectroscopy (FTIR) measurements were conducted using a Magna-IR spectrometer-50 (Nicolet, Thermo Fisher Scientific, San Jose, CA, USA) instrument, employing the conventional KBr pellet technique with a sample-to-KBr ratio of 1:100. The Raman spectrometer (Labram-010, HORIBA Jobin Yvon, Paris, France) employed a solid-state laser (excitation at 532 nm, 35 mW) at room temperature in the range of 200~2000 cm^−1^. The XRD patterns were obtained at room temperature on an Ultimate IV (Rigaku Corporation, Tokyo, Japan) using a Cu target (λ = 0.154 nm) with a 2θ range of 5–80° at a scan rate of 5°/min. The pH values were measured with a pH meter (Leici PHS-3C, Shanghai, China) consisting of a glass electrode.

### 2.3. Preparation of Modified Electrodes

The preparation steps of N-rGO and N-rGO@ppy can be found in the Supplementary Materials. The N-rGO@ppy suspension was prepared by dispersing 6 mg of N-rGO@ppy in 2 mL of ultrapure water, followed by sonication for 30 min, to facilitate the subsequent modifications of GCE and SPE. GCE was sequentially polished using 1.0 μm, 0.3 μm, and 0.05 μm α-alumina powder, thoroughly rinsed with ultrapure water after each polishing step, subsequently sonicated in absolute ethanol and ultrapure water sequentially, and finally dried at room temperature. After this, N-rGO@ppy/GCE was prepared by depositing 20 μL droplets of N-rGO@ppy suspension onto the surface of GCE, followed by drying at room temperature. N-rGO/GCE and ppy/GCE were prepared using the same method. In addition, N-rGO@ppy/SPE was prepared without polishing, and the WE region was modified using droplets of the same concentration and volume of N-rGO@ppy suspension as described above and dried at room temperature.

### 2.4. Electrochemical Analysis Procedure with N-rGO@ppy/GCE

The electrochemical analysis experiments using N-rGO@ppy/GCE were conducted at 25 °C in a quartz electrolyzer (10 mL) without deoxidation to simulate the real-world analysis environment. SWASV measurements involved a three-electrode system immersed in a 0.1 M HAc-NaAc buffer solution containing Pb^2+^, Cd^2+^, and Bi^3+^. The heavy metal ions were pre-enriched at the deposition potential, and then a forward square-wave voltammetric scan was performed. The optimal parameters are as follows: a deposition potential of −1.2 V, a deposition time of 600 s, a scanning range from −1.2 V to −0.2 V, a frequency of 25 Hz, a step increment of 4 mV, and an amplitude of 25 mV. In addition, we applied cyclic voltammetry (CV) to analyze the electrochemical behavior of different electrodes in a 0.1 M KCl solution containing 5 mM [Fe(CN)_6_]^3−/4−^, with the scanning range set from −0.2 V to 0.6 V. The electrolyte solution used for electrochemical impedance spectroscopy (EIS) measurements was the same as CV, with the starting potential as the open circuit potential (OCP), an amplitude of 5 mV, and a frequency range set from 0.01 Hz to 10^6^ Hz.

### 2.5. Sample Pretreatment

Drinking water was collected from the laboratory and acidified to pH 4.5 with HAc. The milk and honey samples were purchased from a supermarket in Yangpu District, Shanghai, China. Milk samples were pretreated according to the existing reports [23] by adding 50 μL of H_2_O_2_ (30 wt.%) to 20 mL of milk samples and sonicated for 15 min, followed by 5 mL of HAc (50 wt.%) and 5 mL of HCl (37 wt.%) for 8 min. The milk mixture samples were then centrifuged at 8000 r/min for 10 min, and the resulting supernatant was filtered through a 0.22 μm Millipore membrane. Finally, the pH was adjusted to 4.5 using an appropriate amount of NaOH solution, and the solution was diluted to a final volume of 40 mL using ultrapure water. To analyze Pb^2+^ and Cd^2+^ concentrations in honey, approximately 2 mL of honey was carefully collected using a disposable pasteurized pipette. Subsequently, the honey sample was subjected to digestion by dissolving it in 5 mL of H_2_O_2_ (30 wt.%) and 2 mL of HNO_3_ (65 wt.%). This resultant mixture was then processed in a microwave oven to facilitate decomposition and filtered through a 0.22 μm Millipore membrane. The pH of the solution was finally adjusted to 4.5 and diluted with ultrapure water to achieve a total volume of 40 mL [24].

### 2.6. Real Sample Analysis with Modified Commercial SPE (N-rGO@ppy/SPE)

The deposition potential and time for N-rGO@ppy/SPE were optimized, establishing the optimal deposition potential at −1.4 V and a deposition time of 300 s. With these optimized experimental conditions, SWASV measurements were conducted on standard samples of Pb^2+^ and Cd^2+^ across various concentration gradients using N-rGO@ppy/SPE. Standard curves correlating the concentrations of heavy metal ions to their respective peak current values were subsequently established. Following this, pretreated samples of drinking water, milk, and honey were analyzed by SWASV using the standard addition method, with N-rGO@ppy/SPE over a scanning range of −1.4 V to −0.4 V. The results obtained were then used to calculate recoveries by referencing the standard curve.

## 3. Results and Discussion

### 3.1. Characterization of N-rGO@ppy

**Figure 1:**
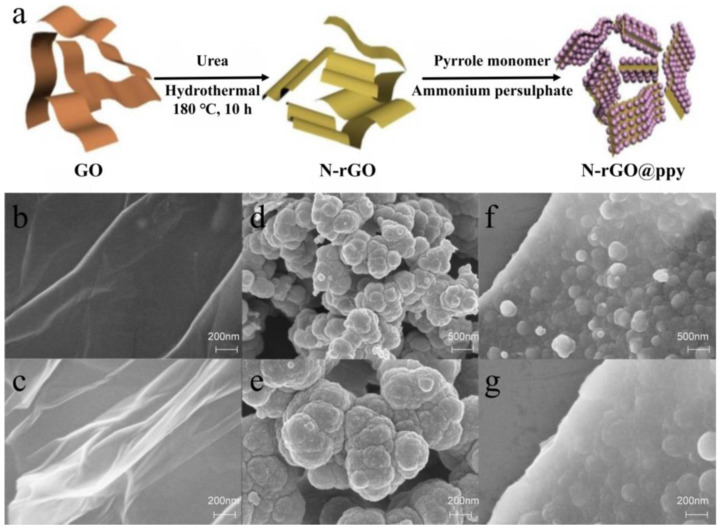
The synthesis procedure of N-rGO@ppy (**a**) and SEM images of GO (**b**), N-rGO (**c**), ppy (**d**,**e**), and N-rGO@ppy (**f**,**g**).

Figure 1a shows the synthesis process of N-rGO@ppy composites. First, GO prepared using the Hummers method has several groups, such as carboxyl (-COOH) and hydroxyl (-OH), significantly improving its water solubility [25]. Then, N-rGO was synthesized hydrothermally using GO and urea as precursors and doped with N atoms. At the same time, most of the oxygen-containing functional groups were reduced, which improved the electrical conductivity of the material and the active sites for heavy metal binding [26]. Finally, in situ polymerization of pyrrole monomer on the surface of N-rGO yielded N-rGO@ppy, which enhanced the stability of the composite and its detection performance for heavy metals. Moreover, the surface structures of the different materials were studied using scanning electron microscopy (SEM), and the results are shown in Figure 1b–g. The morphology of GO (Figure 1b) and N-rGO (Figure 1c) is not significantly different. Both show typical graphene (GR) folds and a smooth surface, with sheets stacked together to form a multilayer structure [27]. The partial agglomeration of N-rGO may be due to the interaction of functional groups, such as via van der Waals forces and hydrogen bonds [28]. Consistent with previous studies, SEM images of chemically polymerized ppy (Figure 1d,e) reveal a distinct aggregate particle morphology. These submicron particles are interconnected, forming several microns in size aggregates, leading to a porous structure with enormous porosity. These structural features may impede efficient electron transfer, affecting ppy’s performance in sensing applications [29,30]. The SEM results of N-rGO@ppy (Figure 1f,g) show that the pyrrole monomer has successfully polymerized along the surface of N-rGO. The increased contact area of ppy with the solution enhances its reactivity, so N-rGO@ppy has a better detection capability for heavy metal ions [31].

**Figure 2:**
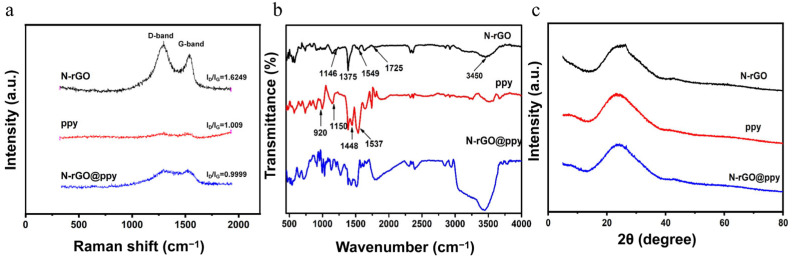
Raman (**a**), FTIR (**b**), and XRD (**c**) spectra of N-rGO, ppy, and N-rGO@ppy.

We used Raman spectroscopy to monitor the structural transformation of N-rGO, ppy, and N-rGO@ppy. As shown in Figure 2a, all Raman spectra have two prominent peaks, at 1350 cm^−1^ and 1580 cm^−1^ [32], consistent with the characteristic D and G bands of the above-mentioned materials. The intensity ratio of the D band to the G band (I_D_/I_G_) can be used to estimate the structural defects of the material. The higher the I_D_/I_G_ value, the higher the defect density [33]. Compared with the I_D_/I_G_ value of N-rGO@ppy (0.9999), the I_D_/I_G_ value of N-rGO increases to 1.6249, which is due to the strong acid oxidation and the introduction of N atoms, leading N-rGO to exhibit more structural defects [34]. In addition, the I_D_/I_G_ values of N-rGO@ppy and ppy indicate that coating the N-rGO surface with ppy masks some of the defects in N-rGO and improves its conductivity. Furthermore, the FTIR results of N-rGO, ppy, and N-rGO@ppy are shown in Figure 2b. The FTIR of N-rGO can identify the main functional groups: -OH stretching at 3450 cm^−1^, attributed to residual moisture in the material, C=O in -COOH at 1725 cm^−1^, and the -OH deformation vibration in CO-H at 1375 cm^−1^ [35]. The absorption peaks at 1549 cm^−1^ and 1146 cm^−1^ are attributed to C=N and C-N, respectively, which confirms that GO was reduced and doped with N atoms under hydrothermal conditions [36]. In the FTIR spectrum of ppy, the characteristic peaks at 1537 cm^−1^ and 1448 cm^−1^ are due to stretching vibrations of symmetric and asymmetric rings. In addition, the characteristic peaks at 1150 cm^−1^ are attributed to the stretching vibration of C-N, and the characteristic peaks at 920 cm^−1^ are attributed to the dipolarization state of ppy [37,38]. The FTIR results of N-rGO@ppy show that the N-rGO@ppy composite material contains the characteristic peaks of N-rGO and ppy, indicating that the ppy molecules can be successfully grafted onto the surface of N-rGO. The crystal structures of the materials were characterized utilizing XRD, with the XRD patterns for N-rGO, ppy, and N-rGO@ppy composites depicted in Figure 2c. The observed diffraction peaks at approximately 2θ = 26.3° (002) and 42.9° (100) in the XRD patterns correspond to N-rGO. Notably, no characteristic peak corresponding to GO was detected around 2θ = 11.4° (001), suggesting the effective reduction of GO under the specified synthesis conditions [39]. Furthermore, the XRD pattern of ppy exhibited a broad characteristic peak in the range of 2θ = 20–30°, which is associated with the amorphous nature of the polymer [19]. In the XRD patterns of the N-rGO@ppy composites, a shift in the diffraction peak from 2θ = 26.3° to 26.6° was observed following the incorporation of ppy (002), which can be attributed to the interaction between the GR lamellae and ppy [40]. Also, this result shows that ppy was successfully grafted onto the surface of N-rGO, which is in agreement with the SEM results.

**Figure 3:**
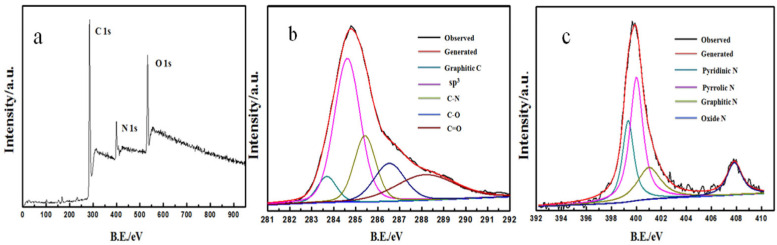
XPS spectrum of N-rGO (**a**); high-resolution XPS data of C1s (**b**) and N1s (**c**).

Numerous studies have demonstrated that doping elements significantly influence the electrochemical properties of carbon-based materials [41,42]. To elucidate the chemical composition of N-rGO, we conducted an X-ray photoelectron spectroscopy (XPS) analysis. Figure 3 illustrates the XPS spectrum of N-rGO, with the corresponding data presented in Table S1. In Figure 3a, the three principal peaks corresponding to C1s, N1s, and O1s at approximately 285.08 eV, 399.08 eV, and 533.08 eV, respectively, are observable, indicating the presence of carbon, oxygen, and N elements within the sample [43]. Furthermore, the high-resolution C1s XPS spectrum of N-rGO, depicted in Figure 3b, can be deconvoluted into five distinct peaks, at 284.1 eV (attributed to graphite C), 284.7 eV (sp^3^ C), 285.6 eV (C-N), 286.6 eV (C-O), and 288.1 eV (C=O) [44]. The N1s high-resolution spectrum, as shown in Figure 3c, reveals four peaks, at 399.4 eV, 400.1 eV, 401.2 eV, and 407.8 eV, corresponding to pyridinic N, pyrrolic N, graphitic N, and oxide N, respectively. The above results confirm the successful incorporation of N atoms into the GO framework [45].

### 3.2. Electrochemical Behavior of GCEs

**Figure 4:**
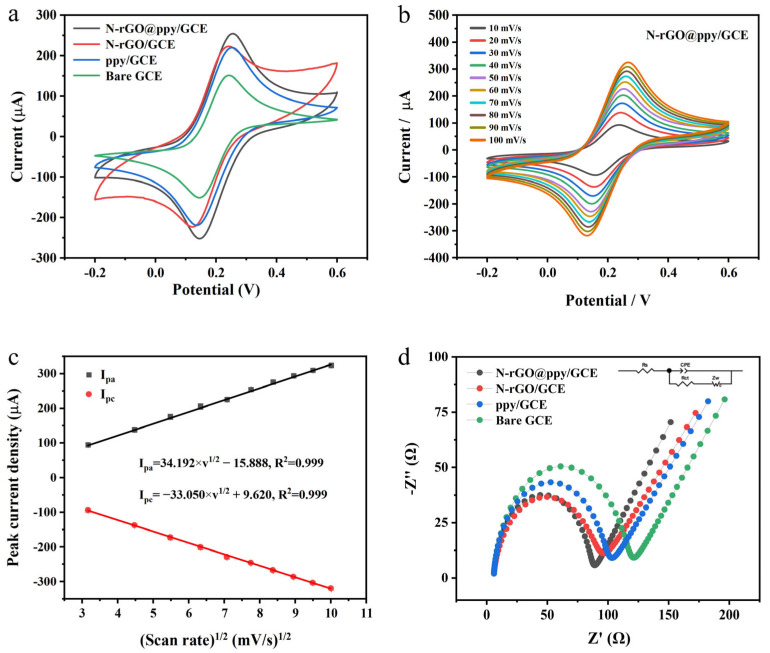
Electrochemical characterization of unmodified and modified GCEs. (**a**) CVs of bare GCE, N-rGO/GCE, ppy/GCE, and N-rGO@ppy/GCE in 0.1 M KCl solution containing 5 mM [Fe(CN)_6_]^3−/4−^; (**b**) CVs of N-rGO@ppy/GCE at 10–100 mV/s; (**c**) oxidation and reduction peak currents as a function of scan rate; (**d**) EIS of different electrodes with the same test solution as for CV.

To investigate the electrochemical activity of the N-rGO@ppy composite material, we modified bare GCEs to create several distinct modified electrodes: N-rGO/GCE, ppy/GCE, and N-rGO@ppy/GCE. CV was conducted in a 0.1 M KCl solution containing 5 mM [Fe(CN)_6_]^3−/4−^ at a scan rate of 50 mV/s, with the results illustrated in Figure 4a. The CV curves for bare GCE (green), ppy/GCE (blue), N-rGO/GCE (red), and N-rGO@ppy/GCE (black) all display a pair of reversible redox peaks. Notably, both ppy/GCE (blue) and N-rGO/GCE (red) exhibited enhanced current responses compared to the bare GCE (green). This improvement is likely attributable to the large specific surface area and favorable electronic properties of the ppy on the electrode surface, alongside the excellent conductivity and rapid charge transfer rate of the N-rGO film [46,47]. As anticipated, N-rGO@ppy/GCE (black) demonstrated the most pronounced redox peak current, indicating that the synergistic combination of N-rGO and ppy significantly enhances the redox performance compared to either material alone. According to the Randles–Sevcik equation,

I_p_ = (2.69 × 10^5^)*n*^3/2^*ACD*^1/2^*ν*^1/2^ (1)

where I_p_ represents the peak current (A), *n* indicates the number of transferred electrons (n = 1 in this case), *A* is the active surface area of the electrode (cm^2^), *C* is the concentration of the reactant (mol/cm^3^), *D* is the diffusion coefficient (cm^2^/s), and *ν* is the scan rate (V/s) [48].

As a result, the electroactive surface areas of N-rGO@ppy/GCE, N-rGO/GCE, ppy/GCE, and bare GCE were calculated to be 0.241, 0.211, 0.208, and 0.143 cm^2^, respectively. This result indicates that the electrochemical reactivity of N-rGO@ppy/GCE exceeds that of the bare GCE, N-rGO/GCE, and ppy/GCE. Therefore, we chose N-rGO@ppy/GCE for electrochemical kinetic testing. The cyclic voltammograms (CVs) of this modified electrode at scan rates ranging from 10 to 100 mV/s are shown in Figure 4b. The redox peak current of N-rGO@ppy/GCE increased with the scan rate, demonstrating a linear relationship between the scan rate and the corresponding anodic or cathodic peak current. The linear fitting results are illustrated in Figure 4c, suggesting that the redox reaction occurring on the surface of N-rGO@ppy/GCE is diffusion-controlled [49].

On the other hand, EIS is frequently utilized to assess the electron transfer characteristics of various electrode surfaces. The diameter of the observed impedance semicircle is indicative of the charge transfer resistance (Rct), where an elevated Rct can significantly hinder the electrochemical processes occurring at the electrode/solution interface [50]. Figure 4d illustrates the Nyquist plots for various electrodes, including bare GCE (green), ppy/GCE (blue), N-rGO/GCE (red), and N-rGO@ppy/GCE (black). The measurements were conducted in the identical test solution utilized for CV, with a starting potential of OCP, an amplitude of 5 mV, and a frequency range of 0.01 to 10^6^ Hz. At higher frequencies, bare GCE presents a semicircle of approximately 116 Ω in diameter, indicating the diffusion-limited step of the electrochemical process. After surface modification with N-rGO and ppy, the semicircle diameters decrease to approximately 89 Ω and 97 Ω, respectively. This result suggests that both N-rGO and ppy substantially enhance electron transfer kinetics. Among the electrodes tested, the N-rGO@ppy/GCE shows the smallest Rct, with a semicircle diameter further decreased to around 82 Ω, which suggests that there is a good synergistic effect between N-rGO and ppy, leading the N-rGO@ppy composites to exhibit superior electronic conduction properties. The electrochemical results obtained from EIS are consistent with the prior findings from CV, thereby confirming the successful modification of the GCE surface with the N-rGO@ppy composite material.

### 3.3. Optimization of Electrochemical Detection Parameters for N-rGO@ppy/GCE

Several parameters were carefully optimized to enhance the performance of N-rGO@ppy/GCE for the simultaneous detection of Pb^2+^ and Cd^2+^. As shown in Figure S1, the following factors influenced the SWASV dissolution peak currents of Pb^2+^ and Cd^2+^: (a) deposition time, (b) Bi^3+^ concentration, (c) Nafion concentration, (d) dosage of the N-rGO@ppy composite, (e) deposition potential, and (f) pH. Based on the experimental results, the optimal conditions for detection in a 0.1 M HAc-NaAc buffer were determined to be a deposition potential of −1.2 V, a deposition time of 600 s, a Bi^3+^ concentration of 500 μg L^−1^, a Nafion concentration of 0.05 wt.%, an N-rGO@ppy composite dosage of 3 mg mL^−1^, and a solution pH of 4.5. Specific discussions regarding these six parameters can be found in the Supplementary Materials.

### 3.4. Analytical Performance of GCEs for Simultaneous Detection of Cd^2+^ and Pb^2+^

**Figure 5:**
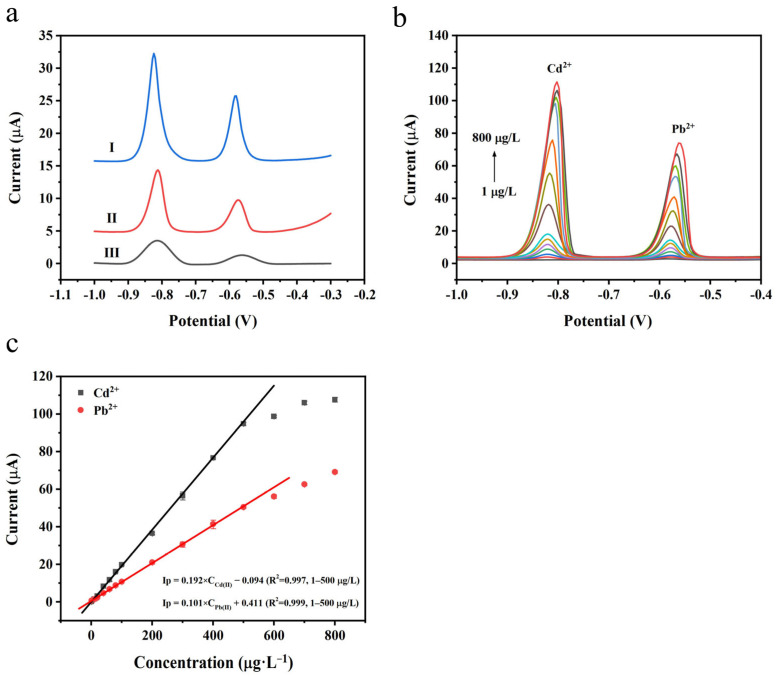
(**a**) SWASVs of 100 μg L^−1^ Pb^2+^ and Cd^2+^ on N-rGO@ppy/GCE (curve I), ppy/GCE (curve II), and N-rGO/GCE (curve III) in 0.1 M HAc-NaAc buffer (pH = 4.5); (**b**) SWASVs of simultaneous detection of Pb^2+^ and Cd^2+^ at different concentrations on N-rGO@ppy/GCE; (**c**) calibration curves for concentrations of 1–500 μg L^−1^.

Under optimized SWASV experimental conditions, we compared the performances of N-rGO/GCE, ppy/GCE, and N-rGO@ppy/GCE in the simultaneous detection of Pb^2+^ and Cd^2+^, and the results are shown in Figure 5a. Pb^2+^ and Cd^2+^ demonstrate lower dissolution peaks on N-rGO/GCE (Figure 5a, curve III), likely due to the limited dispersibility and conductivity of N-rGO on the electrode surface. In contrast, in Figure 5a, curve II illustrates that ppy/GCE has a more pronounced stripping peak. However, its peak current value remains lower than that of N-rGO@ppy/GCE (Figure 5a, curve I), which can be attributed to the agglomeration of ppy, diminishing the adsorption sites for heavy metal ions. The enhanced electrochemical response signal for Pb^2+^ and Cd^2+^ observed on N-rGO@ppy/GCE may be ascribed to four key factors. First, N-rGO@ppy exhibits a strong affinity for heavy metal ions due to specific functional groups such as -NH_2_ and -COOH within its structure. Second, an increase in active sites leads to an improved adsorption rate. The polymerization of pyrrole monomers on N-rGO helps minimize the agglomeration of ppy, thereby revealing more adsorption sites. Third, the heat treatment-induced expansion of N-rGO@ppy significantly mitigates π-π stacking, enhancing the modified material’s pore volume and specific surface area. Lastly, the better dispersibility and conductivity of N-rGO@ppy can be attributed to the synergistic impact of the N atoms doped into the structure and ppy, which further accelerates charge transfer at the electrode [51]. Consequently, N-rGO@ppy/GCE can be regarded as a high-performance sensing platform for detecting Pb^2+^ and Cd^2+^, offering high sensitivity and a pronounced stripping peak current.

Then, we conducted SWASV experiments under optimal test conditions to investigate the performance of N-rGO@ppy/GCE for the simultaneous detection of Pb^2+^ and Cd^2+^. Figure 5b,c illustrates the square-wave voltammetric responses of Pb^2+^ and Cd^2+^ at various concentrations, along with the corresponding calibration curves. In Figure 5b, the peaks for Pb^2+^ and Cd^2+^ can be observed at potentials of approximately −0.58 V and −0.82 V, respectively. The peak currents are directly proportional to the concentrations of Pb^2+^ and Cd^2+^. In Figure 5c, the regression equations for the concentration range of 1–500 μg L^−1^ are y = 0.101x + 0.411 (R^2^ =0.999) for Pb^2+^ and y = 0.192x − 0.094 (R^2^ = 0.997) for Cd^2+^ (y is peak current, µA; x represents Pb^2+^ and Cd^2+^ concentrations, μg L^−1^; the number of data points is 11). According to the 3σ/slope method (wherein σ denotes the standard deviation of the blank samples) [52], the LODs for Pb^2+^ and Cd^2+^ were calculated to be 0.080 μg L^−1^ and 0.029 μg L^−1^, respectively. Table 1 compares the sensor investigated in this study with previously reported modified electrodes for detecting Pb^2+^ and Cd^2+^, and it is clear that N-rGO@ppy/GCE demonstrates a comparable or even superior analytical performance.

### 3.5. Interference Resistance, Stability, and Repeatability of N-rGO@ppy/GCE

Experiments were conducted to evaluate the interference resistance of N-rGO@ppy/GCE by introducing interfering metal ions, including Ca^2+^, Zn^2+^, Mg^2+^, Mn^2+^, Fe^3+^, and Sn^2+^, with 10 times the concentration of target metal ions (Pb^2+^ and Cd^2+^), considering the potential co-deposition of other metal ions with Pb^2+^ and Cd^2+^, which may affect the determination. According to the results presented in Figure S1a, the influence of interfering metal ions on the peak currents of Pb^2+^ and Cd^2+^ is minimal, recorded at less than 3.8% and 2.2%, respectively. Additionally, the stability of N-rGO@ppy/GCE was evaluated over a ten-day period, as depicted in Figure S1b. This assessment involved performing three parallel measurements on the day of application of the N-rGO@ppy drop-coating modification and subsequently on days 2, 4, 6, 8, and 10. The stripping peak currents for Pb^2+^ and Cd^2+^ show only minor fluctuations, with relative standard deviations (RSDs) of 6.0% for Pb^2+^ and 3.3% for Cd^2+^. In addition, the repeatability of the modified electrodes was assessed in solutions containing 100 μg L^−1^ of Pb^2+^ and Cd^2+^ on the same day in two different aspects: Figure S1c presents the results of 10 consecutive measurements using the same N-rGO@ppy/GCE, showing an RSD of 2.7% for Pb^2+^ and 1.0% for Cd^2+^. Figure S1d displays the results of three repeated measurements conducted by the same analyst with each of the five newly prepared N-rGO@ppy/GCEs, resulting in an RSD of 2.8% for Pb^2+^ and 1.4% for Cd^2+^.

### 3.6. Practical Application of N-rGO@ppy/SPE

**Figure 6:**
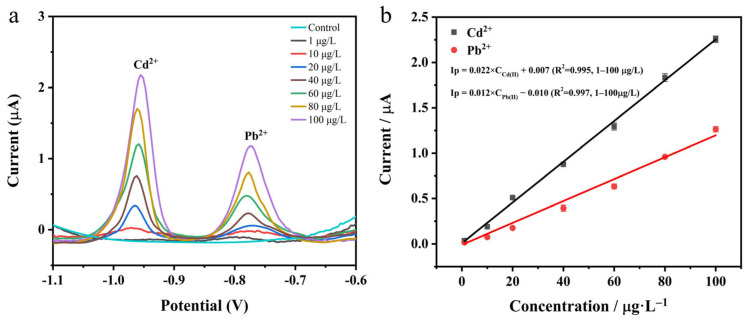
(**a**) SWASVs of simultaneous detection of Pb^2+^ and Cd^2+^ at different concentrations on N-rGO@ppy/SPE; (**b**) calibration curves for concentrations of 1–100 μg L^−1^.

To address the limitations of the traditional three-electrode system for in situ detection, we modified the commercially available SPE with the synthesized N-rGO@ppy composite to further validate the feasibility of our proposed sensing strategy. We optimized the deposition potential and time for N-rGO@ppy/SPE in the context of SWASV measurements, as illustrated in Figure S3. Upon a comprehensive evaluation of detection sensitivity and time efficiency, the optimal deposition potential was established at −1.4 V, with a corresponding deposition duration of 300 s. The SWASV analysis was conducted under these optimized conditions using a mixture of trace (1 µg L^−1^) and higher-concentration (100 µg L^−1^) solutions of Pb^2+^ and Cd^2+^, presented in Figure S4. The square-wave voltammetric responses of Pb^2+^ and Cd^2+^ at varying concentrations are illustrated in Figure 6, accompanied by the corresponding calibration curves. Distinct stripping peaks for Pb^2+^ and Cd^2+^ can be observed at potentials of approximately −0.78 V and −0.96 V, respectively, as shown in Figure 6a. The peak current values exhibit a direct proportionality to the respective ion concentrations. In Figure 6b, the regression equations for the concentration range of 1–100 µg L^−1^ are y = 0.012x − 0.010 (R^2^ = 0.997) for Pb^2+^ and y = 0.022x + 0.007 (R^2^ = 0.995) for Cd^2+^ (y is peak current, µA; x represents Pb^2+^ and Cd^2+^ concentrations, µg L^−1^; the number of data points is 7). The LODs were determined to be 0.662 µg L^−1^ for Pb^2+^ and 0.470 µg L^−1^ for Cd^2+^ (3σ/slope). While the sensitivity of N-rGO@ppy/SPE for the detection of Pb^2+^ and Cd^2+^ was observed to be slightly diminished in comparison to that of N-rGO@ppy/GCE, both electrodes underscore the promising applicability of N-rGO@ppy composites for the in situ detection of heavy metals. In the analysis of drinking water, milk, and honey samples, characteristic peaks indicative of Pb^2+^ and Cd^2+^ were not observed in the pretreated samples, which did not contain added heavy metals, when using N-rGO@ppy/SPE. However, when trace amounts of the target metal ions were introduced through the standard addition method [62], distinct dissolved peaks were identified upon re-analysis with N-rGO@ppy/SPE. For validation purposes, graphite furnace atomic absorption spectrometry (GFAAS) was employed as the reference method. The experimental results showed no statistically significant difference between the two analytical techniques, and the recoveries obtained using N-rGO@ppy/SPE were satisfactory (refer to Table S2).

## 4. Conclusions

This study introduces a novel electrochemical sensing strategy utilizing N-rGO@ppy composite-modified electrodes for the simultaneous trace detection of Pb^2+^ and Cd^2+^. The composite cannot only effectively solve the agglomeration problem of ppy but also enhances heavy metal ion trapping capabilities. Under the optimal conditions, N-rGO@ppy/GCE exhibits a wide linear working range (1–500 μg L^−1^) with LODs of 0.080 μg L^−1^ for Pb^2+^ and 0.029 μg L^−1^ for Cd^2+^, along with impressive interference resistance, stability, and repeatability. In addition, the N-rGO@ppy composite significantly enhances the electrochemical performance of commercial SPE, with the LODs for modified SPE for Pb^2+^ and Cd^2+^ found to be 0.662 μg L^−1^ and 0.470 μg L^−1^, respectively. When using GFAAS as a reference method, the trace analysis results of N-rGO@ppy/SPE and GFAAS for Pb^2+^ and Cd^2+^ in three samples—drinking water, milk, and honey—showed no significant differences. The electrochemical sensors developed in this study offer advantages such as cost-effectiveness, portability, potential for miniaturization, and suitability for in situ monitoring, representing a powerful complement or alternative to traditional analytical techniques with promising applications in food analysis and environmental monitoring. However, this study does have limitations. The pretreatment process for food samples is similar to traditional digestion procedures used in spectroscopic analysis (such as AAS and ICP-MS), which involve oxidizing agents like H_2_O_2_ and strong acids such as concentrated HCl, limiting the practicality of N-rGO@ppy/SPE. Moreover, it must be mentioned that the complex matrices of food samples, such as those found in milk and honey, still pose significant challenges for developing reliable analytical methods. Future research could focus on several areas, such as developing anti-fouling coatings like polyethene glycol to prevent non-specific adsorption and exploring more efficient pretreatment methods tailored to specific sample types.

## Figures and Tables

**Table 1:** Comparison of the analytical performance of N-rGO@ppy/GCE and other electrodes for Pb^2+^ and Cd^2+^.

Electrode	Technique	Metal Ions	Linear Ranges (μg L^−1^)	Detection Limits (μg L^−1^)	References
PA/PPy/GO/GCE	DPASV	Cd^2+^	5–150	2.13	[53]
		Pb^2+^	5–150	0.41	
Nafion/Bi/NMC/GCE	DPASV	Cd^2+^	2–100	1.5	[54]
		Pb^2+^	0.5–100	0.05	
ERGNO/BiF/SPE	SWASV	Cd^2+^	1–60	0.5	[55]
		Pb^2+^	1–60	0.8	
GO-Fe_3_O_4_-PAMAM/GCE	SWASV	Cd^2+^	0.2–140	0.07	[56]
		Pb^2+^	0.4–120	0.13	
GSH@Fe_3_O_4_/MGCE	SWASV	Cd^2+^	0.5–100	0.171	[57]
		Pb^2+^	0.5–100	0.182	
BiNPs@NPCGSc/GCE	SWASV	Cd^2+^	9–90	0.5	[58]
		Pb^2+^	12–124	0.7	
NiFe_2_O_4_/PPy/GCE	SWASV	Pb^2+^	21–435	0.8	[59]
3DGO-Py10/GCE	SWASV	Cd^2+^	5–400	3.6	[60]
PA-doped PPy/MoS_2_/GCE	DPASV	Cd^2+^	10–300	2.03	[61]
		Pb^2+^	10–300	1.78	
N-rGO@ppy/GCE	SWASV	Cd^2+^	1–500	0.029	This work
		Pb^2+^	1–500	0.080	

PA: phytic acid; PPy: polypyrrole; GO: graphene oxide; GCE: glassy carbon electrode; DPASV: differential-pulse anodic stripping voltammetry; NMC: nitrogen-doped microporous carbon; ERGNO: electrochemically reduced graphene oxide; BiF: bismuth film; SPE: screen-printed electrode; SWASV: square-wave anodic stripping voltammetry; PAMAM: poly(amidoamine); GSH: glutathione; MGCE: magnetic glassy carbon electrode; BiNPs: bismuth nanoparticles; NPCGSc: enriched nanoporous carbon on graphene sheet; 3DGO: three-dimensional graphene oxide.

## Data Availability

The data presented in this study are available upon request from the corresponding author.

## Supplementary Materials

The following supporting information can be downloaded at https://www.mdpi.com/article/10.3390/bios15050267/s1, Figure S1: Effect of deposition time (a), Bi^3+^ concentration (b), Nafion concentration (c), dosage of N-rGO@ppy composite (d), deposition potential (e), and pH (f) in 0.1 M HAc-NaAc buffer solution on the peak current of SWASV for 100 μg L^−1^ Cd^2+^ and 100 μg L^−1^ Pb^2+^; Figure S2: (a) SWASV peak current of N-rGO@ppy/GCE in 0.1 M HAc-NaAc solutions containing 100 μg L^−1^ Pb^2+^ and Cd^2+^ and in the presence of 1 mg L^−1^ Ca^2+^, Zn^2+^, Mg^2+^, Mn^2+^, Fe^3+^, and Sn^2+^ interfering metal ions (n = 3). (b) Stability of N-rGO@ppy/GCE for 10 days (n = 3). (c) Ten continuous detections of the same N-rGO@ppy/GCE. (d) The detection of five different N-rGO@ppy/GCE (n = 3); Figure S3: Effect of (a) deposition potential and (b) deposition time in 0.1 M HAc-NaAc buffer solution on the peak current of SWASV for 100 μg L^−1^ Cd^2+^ and 100 μg L^−1^ Pb^2+^; Figure S4: (a) Dripping N-rGO@ppy composite liquid droplets to modify commercially available SPE; unsmoothed SWASVs of Pb^2+^ and Cd^2+^ at a concentration of (b) 1 μg L^−1^ or (c) 100 μg L^−1^ obtained using N-rGO@ppy/SPE in 0.1 M HAc-NaAc buffer (pH = 4.5); Table S1: C, O, and N atomic percentages of N-rGO measured by XPS; Table S2: Comparison of SWASV responses for N-rGO@ppy/SPE and GFAAS in analyzing Pb^2+^ and Cd^2+^ levels in drinking water, milk, and honey samples. References [63,64,65,66,67,68] are cited in the Supplementary Materials.
