# Supplementary material for: Simultaneous Trace Analysis of Lead and Cadmium in Drinking Water, Milk, and Honey Samples Through Modified Screen-Printed Electrode
Source: Biosensors (Basel). 2025 Apr 23;15(5):267. doi: 10.3390/bios15050267 (PMC12109976; doi:10.3390/bios15050267)
Supplement: Supplementary file 1 [file biosensors-15-00267-s001.zip › biosensors-3520357-supplementary.pdf]

# Supplementary material

## 1. Preparation of N-rGO

Graphene oxide (GO) was synthesized using a modified Hummers method [63]. 60 mg of GO was initially dispersed in 60 mL of ultrapure water and sonicated for 3 h. Subsequently, 300 mg of urea was gradually incorporated into the solution. This mixture was then transferred to a 100 mL hydrothermal reactor and heated at 180 ℃ for 10 h. The resulting product was centrifuged, thoroughly washed with ethanol and ultrapure water, and then dried in a vacuum oven at 60 ℃ for 24 h to yield N-rGO.

## 2. Preparation of N-rGO@ppy

A suspension of 50 mg of N-rGO was prepared by dispersing the material in 100 mL of ultrapure water within a round-bottom flask. This mixture was subjected to sonication for 2 h to ensure uniform dispersion. Subsequently, 100 μL of pyrrole (Py) was introduced into the N-rGO suspension, and sonication was continued for an additional 10 min to promote the interaction between the components. A solution containing 400 mg of ammonium persulfate (APS) was prepared in 40 mL of ultrapure water. The APS solution was then gradually added to the Py/N-rGO mixture while maintaining the reaction under an ice bath for 12 h. Upon completion of the polymerization, N-rGO@ppy was obtained by centrifugation, followed by thorough washing and subsequent drying of the resultant product.

## 3. XPS data of N-rGO

Table S1. C, O, and N atomic percentages of N-rGO measured by XPS.

| Element | Atomic percentage (%) |
|---|---|
| C | 81.1 |
| O | 14.19 |
| N | 4.71 |

## 4. Optimization of electrochemical detection parameters for N-rGO@ppy/GCE

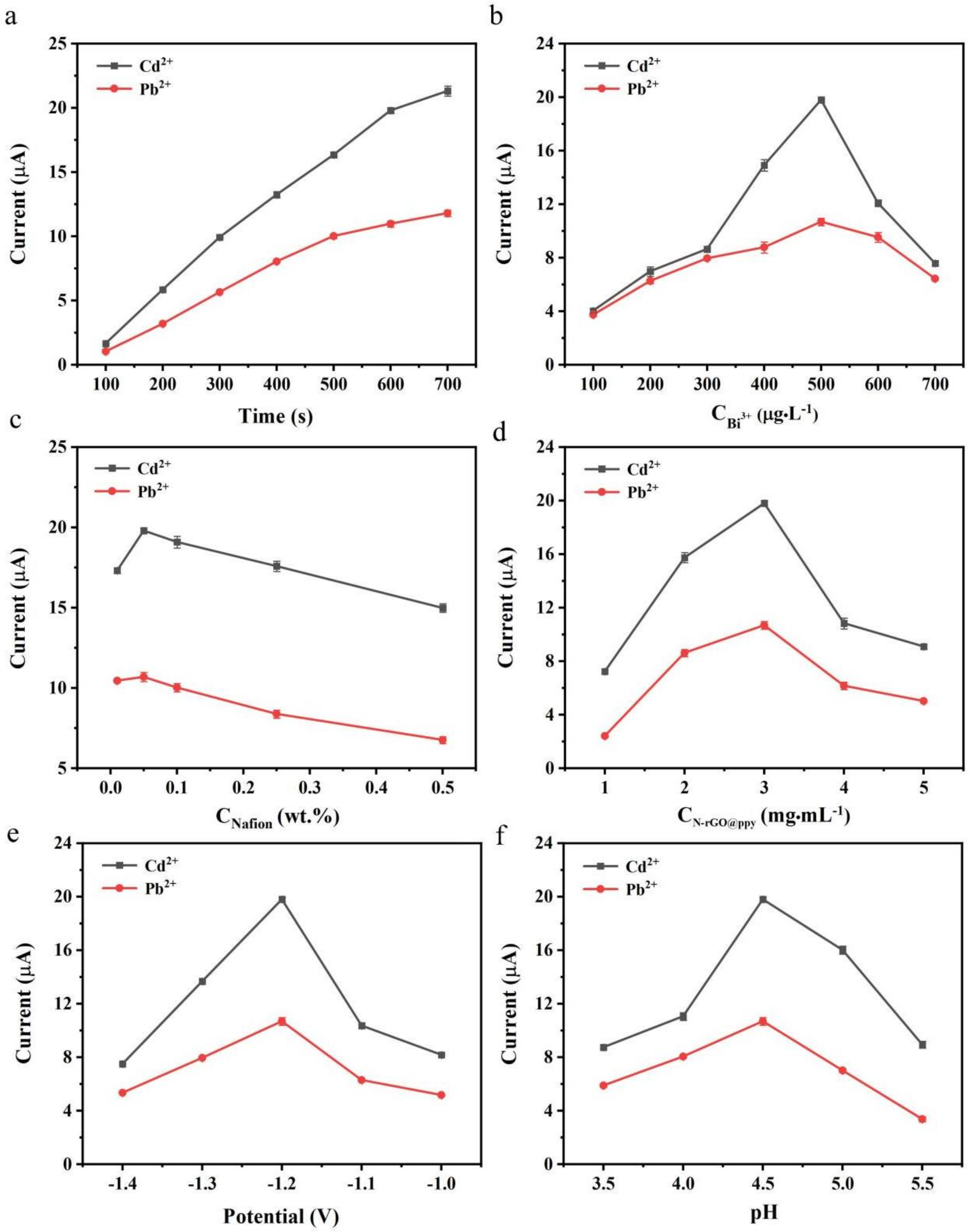


Figure S1. Effect of deposition time (a), $Bi^{3+}$ concentration (b), Nafion concentration (c), dosage of N-rGO@ppy composite (d), deposition potential (e), and pH (f) in 0.1 M HAc-NaAc buffer solution on the peak current of SWASV for 100 μg $L^{-1}$ $Cd^{2+}$ and 100 μg $L^{-1}$ $Pb^{2+}$.

Figure. S1a illustrates the impact of deposition time on heavy metal ions' anodic dissolution peak current, with concentrations of 100 μg $L^{-1}$ for $Cd^{2+}$ and $Pb^{2+}$. This experiment utilized a $Bi^{3+}$ concentration of 500 μg $L^{-1}$ and a Nafion concentration of 0.05

wt.% alongside a 3 mg $mL^{-1}$ N-rGO@ppy composite material, with the deposition potential maintained at -1.2 V and under pH 4.5. The data indicates that with an increase in deposition time, there is a corresponding rise in the peak currents for both $Pb^{2+}$ and $Cd^{2+}$, suggesting that prolonged deposition periods enhance the accumulation of these heavy metal ions on the surface of the N-rGO@ppy/GCE. Notably, after a deposition time of 600 s, the peak currents for $Pb^{2+}$ and $Cd^{2+}$ approach saturation levels. Consequently, a deposition time of 600 s has been determined as optimal for subsequent experimental investigations.

The stripping performance of the N-rGO@ppy/GCE can be further enhanced by in situ formation of bismuth (Bi) film on the electrode surface. Unlike the toxic mercury electrode, the Bi film facilitates the acquisition of distinct and well-defined stripping peaks. As illustrated in Figure. S1b, the peak currents for $Pb^{2+}$ and $Cd^{2+}$ initially increase and then decrease with an increase in $Bi^{3+}$ concentration. The other parameters are set at 100 μg $L^{-1}$ for both $Cd^{2+}$ and $Pb^{2+}$, a Nafion concentration of 0.05 wt.%, a 3 mg $mL^{-1}$ concentration of N-rGO@ppy composite, a deposition potential of -1.2 V, a pH of 4.5, and a deposition time of 600 s. When the $Bi^{3+}$ concentration exceeds 500 μg $L^{-1}$, the observed decline in peak current is likely due to the formation of a thick Bi film during the stripping process, which hinders the mass transfer of $Pb^{2+}$ and $Cd^{2+}$ [64]. For this reason, a $Bi^{3+}$ concentration of 500 μg $L^{-1}$ was selected for subsequent experiments.

The subsequent parameter optimization methodology mirrors the previously described optimization process; the remaining detection parameters are maintained in their optimal state while individual parameters are optimized. As depicted in Figure. S1c, the ideal modification concentration of Nafion was determined to be 0.05 wt.%. Nafion is extensively utilized for electrode modification, attributed to its distinctive antifouling properties, excellent film-forming capabilities, and substantial adsorption potential [65]. An increase in Nafion concentration from 0.01 wt.% to 0.05 wt.% enhanced the peak currents of $Pb^{2+}$ and $Cd^{2+}$. This effect can be ascribed to the hydrophilic, negatively charged nature of the Nafion structure, which facilitates the selective accumulation of positively charged heavy metal ions through electrostatic interactions. However, excessive Nafion application may lead to adverse effects, including impediments to ion transport and diminished electrode conductivity [66]. Therefore, a Nafion concentration of 0.05 wt.% was deemed optimal for subsequent investigations.

Utilizing the active sites of the material for the adsorption of heavy metal ions is essential for enhancing detection sensitivity. As shown in Figure. S1d, the effect of varying amounts of N-rGO@ppy on the electrode's stripping response indicates that within the concentration range of 0-3 mg $mL^{-1}$, the peak current increases with the concentration of N-rGO@ppy. This increase can be attributed to the modification of the N-rGO@ppy composite material, which enhances the number of active sites as well as the conductivity and electron transfer rate. However, when the concentration of N-rGO@ppy exceeds 3 mg $mL^{-1}$, a decline in the peak current for $Pb^{2+}$ and $Cd^{2+}$ dissolution is observed. This decrease may be due to a reduced material utilization per unit mass. Thus, 3 mg $mL^{-1}$ is identified as the optimal dosage of N-rGO@ppy.

As depicted in Figure. S1e, we also investigated the influence of deposition potential on the electrode stripping response. It is evident that as the deposition potential is

negatively shifted from -1.0 V to -1.2 V, the peak current for the dissolution of $Pb^{2+}$ and $Cd^{2+}$ increases. However, with a further negative shift in the deposition potential from -1.2 V to -1.4 V, the peak current for $Pb^{2+}$ and $Cd^{2+}$ dissolution begins to decline. The highest peak dissolution currents of $Pb^{2+}$ and $Cd^{2+}$ were observed at a deposition potential of -1.2 V. This phenomenon can be attributed to the incomplete reduction of $Pb^{2+}$ and $Cd^{2+}$ in solution at more positive potentials and the onset of the hydrogen evolution reaction (HER) at more negative potentials, both of which influence the deposition of heavy metal ions [67]. Consequently, a deposition potential of -1.2 V was selected for further investigation.

The pH of the electrolyte solution plays a crucial role in the electrochemical detection of $Pb^{2+}$ and $Cd^{2+}$. Consequently, we examined the effect of a pH range of 3.5 to 5.5 in a 0.1 M NaAc-HAc buffer on the dissolution peak current. As illustrated in Figure. S1f, the peak currents for $Pb^{2+}$ and $Cd^{2+}$ exhibit a strong dependence on the electrolyte's pH. At pH 3.5, higher concentrations of $H^+$ in solution lead to elevated HER currents, and $H^+$ can compete with the target heavy metal ions for limited deposition sites, resulting in a reduced electrochemical response of the modified electrode to $Pb^{2+}$ and $Cd^{2+}$. However, the response increases rapidly, with the maximum stripping peak current observed at pH 4.5. Beyond this point, from pH 4.5 to 5.5, the peak current decreased significantly, likely due to the propensity of $Pb^{2+}$ and $Cd^{2+}$ to undergo hydrolysis, forming metal hydroxides [68]. Thus, pH 4.5 was identified as the optimal pH for analysis.

## 5. Interference resistance, stability, and repeatability of N-rGO@ppy/GCE

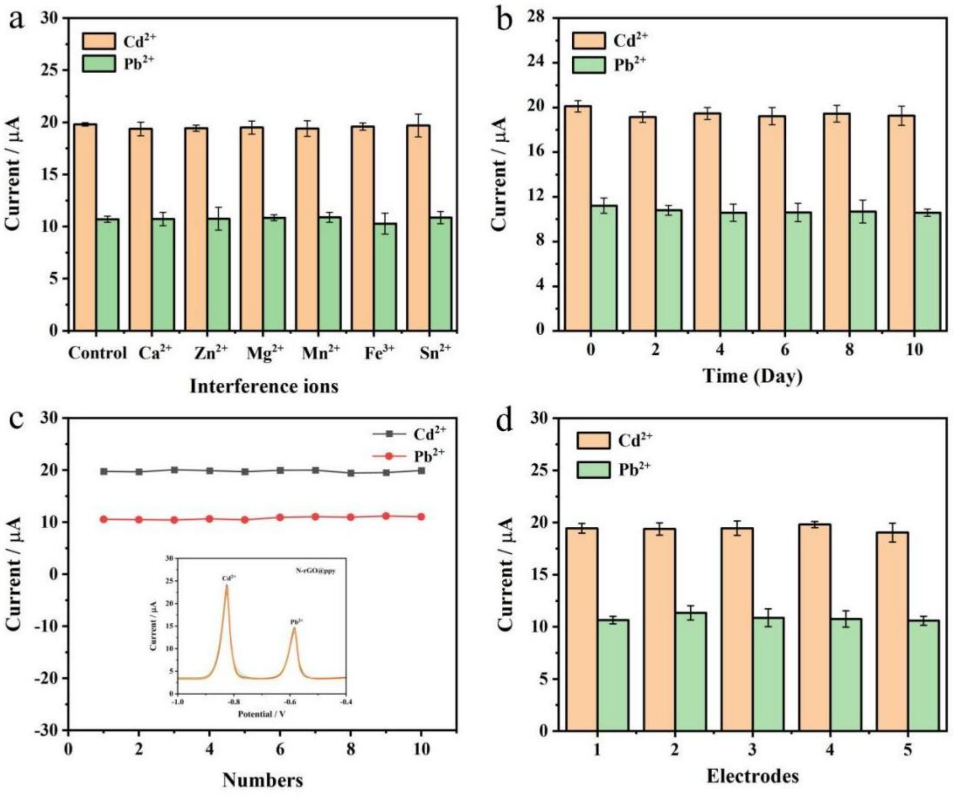


Figure. S2. (a) SWASV peak current of N-rGO@ppy/GCE in 0.1 M HAc-NaAc

solutions containing 100 μg $L^{-1}$ $Pb^{2+}$ and $Cd^{2+}$ and in the presence of 1 mg $L^{-1}$ $Ca^{2+}$, $Zn^{2+}$, $Mg^{2+}$, $Mn^{2+}$, $Fe^{3+}$ and $Sn^{2+}$ interfering metal ions (n = 3). (b) Stability of N-rGO@ppy/GCE for 10 days (n = 3). (c) Ten continuous detections of the same N-rGO@ppy/GCE. (d) The detection of five different N-rGO@ppy/GCE (n = 3).

## 6. Optimization of detection parameters and feasibility validation of N-rGO@ppy/SPE

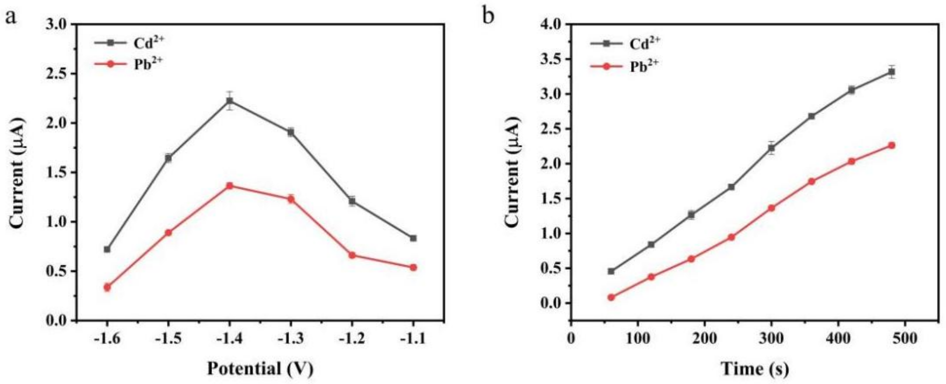


Figure. S3. Effect of (a) deposition potential and (b) deposition time in 0.1 M HAc-NaAc buffer solution on the peak current of SWASV for 100 μg $L^{-1}$ $Cd^{2+}$ and 100 μg $L^{-1}$ $Pb^{2+}$.

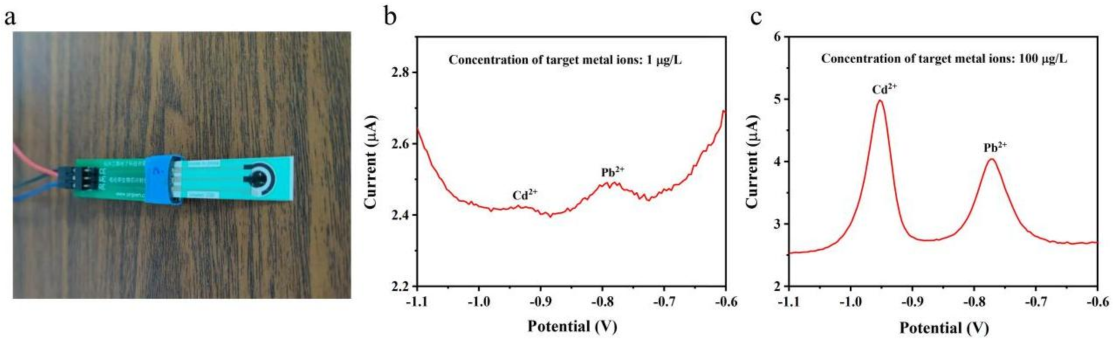


Figure. S4. (a) Dripping N-rGO@ppy composite liquid droplets to modify commercially available SPE; Unsmoothed SWASVs of $Pb^{2+}$ and $Cd^{2+}$ at a concentration of (b) 1 μg $L^{-1}$ or (c) 100 μg $L^{-1}$ obtained using N-rGO@ppy/SPE in 0.1 M HAc-NaAc buffer (pH=4.5).

## 7. Actual sample analysis

Table S2. Comparison of SWASV Responses for N-rGO@ppy/SPE and GFAAS in analyzing $Pb^{2+}$ and $Cd^{2+}$ levels in drinking water, milk, and honey samples.

| Sample | Analytes | GFAAS | | | | N-rGO@ppy/SPE | | | | *t*-values* |
|---|---|---|---|---|---|---|---|---|---|---|
| | | Added (μg $L^{-1}$) | Found (μg $L^{-1}$) | Recovery (%) | RSD (%, n = 3) | Added (μg $L^{-1}$) | Found (μg $L^{-1}$) | Recovery (%) | RSD (%, n = 3) | |

| | | | | | | | | | | |
|---|---|---|---|---|---|---|---|---|---|---|
| Drinking Water | $Cd^{2+}$ | 2 | 2.05 | 102.48 | 2.86 | 2 | 1.97 | 98.52 | 5.22 | 1.171 |
| | | 5 | 4.93 | 98.62 | 3.41 | 5 | 4.90 | 98.05 | 3.84 | 0.206 |
| | | 10 | 10.19 | 101.87 | 2.53 | 10 | 10.28 | 102.82 | 4.42 | 0.298 |
| | $Pb^{2+}$ | 2 | 1.94 | 97.04 | 2.37 | 2 | 2.02 | 101.11 | 4.46 | 1.370 |
| | | 5 | 4.91 | 98.19 | 3.42 | 5 | 4.88 | 97.58 | 3.77 | 0.209 |
| | | 10 | 9.87 | 98.73 | 1.84 | 10 | 9.80 | 98.03 | 1.98 | 0.456 |
| Milk | $Cd^{2+}$ | 2 | 1.89 | 94.42 | 2.79 | 2 | 1.94 | 96.76 | 4.40 | 0.863 |
| | | 5 | 4.76 | 95.18 | 3.12 | 5 | 4.81 | 96.19 | 2.62 | 0.445 |
| | | 10 | 9.43 | 94.26 | 3.98 | 10 | 9.30 | 92.97 | 2.54 | 0.508 |
| | $Pb^{2+}$ | 2 | 1.92 | 96.03 | 3.25 | 2 | 2.03 | 101.47 | 4.51 | 1.720 |
| | | 5 | 4.68 | 93.61 | 4.13 | 5 | 4.59 | 91.76 | 5.37 | 0.498 |
| | | 10 | 9.93 | 99.27 | 1.56 | 10 | 10.10 | 100.98 | 1.73 | 1.261 |
| Honey | $Cd^{2+}$ | 2 | 1.79 | 89.52 | 4.47 | 2 | 1.84 | 92.07 | 5.18 | 0.696 |
| | | 5 | 4.92 | 98.43 | 2.84 | 5 | 4.67 | 93.44 | 3.48 | 1.993 |
| | | 10 | 9.72 | 97.15 | 3.06 | 10 | 9.55 | 95.46 | 2.13 | 0.817 |
| | $Pb^{2+}$ | 2 | 1.84 | 91.96 | 3.82 | 2 | 1.90 | 94.94 | 4.95 | 0.885 |
| | | 5 | 4.97 | 99.38 | 1.95 | 5 | 4.79 | 95.83 | 4.69 | 1.274 |
| | | 10 | 9.55 | 95.47 | 2.68 | 10 | 9.41 | 94.09 | 2.45 | 0.704 |

*: $\alpha = 0.05$, two-tailed test, $df = 4$, and $t_{0.05/2,4} \approx 2.776$.

# References


63. Yu, J., M. Lin, Q. Tan and J. Li. High-value utilization of graphite electrodes in spent lithium-ion batteries: From 3d waste graphite to 2d graphene oxide. *J. Hazard. Mater.* **2021**, *401*, 123715.
64. Arduini, F.; Calvo, J.Q.; Palleschi, G.; Moscone, D.; Amine, A. Bismuth-modified electrodes for lead detection. *TrAC Trends Anal. Chem.* **2010**, *29*, 1295–1304.
65. Yu, L.; Zhang, Q.; Yang, B.; Xu, Q.; Xu, Q.; Hu, X. Electrochemical Sensor Construction Based on Nafion/Calcium Lignosulphonate Functionalized Porous Graphene Nanocomposite and Its Application for Simultaneous Detection of Trace $Pb^{2+}$ and $Cd^{2+}$. *Sens. Actuators B Chem.* **2018**, *259*, 540–551.
66. Pizarro, J.; Segura, R.; Tapia, D.; Navarro, F.; Fuenzalida, F.; Aguirre, M.J. Inexpensive and green electrochemical sensor for the determination of Cd(II) and Pb(II) by square wave anodic stripping voltammetry in bivalve mollusks. *Food Chem.* **2020**, *321*, 126682.
67. Huang, R.; Lv, J.; Chen, J.; Zhu, Y.; Zhu, J.; Wågberg, T.; Hu, G. Three-dimensional porous high boron-nitrogen-doped carbon for the ultrasensitive electrochemical detection of trace heavy metals in food samples. *J. Hazard. Mater.* **2023**, *442*, 130020.
68. Bashir, A.; Malik, L.A.; Ahad, S.; Manzoor, T.; Bhat, M.A.; Dar, G.; Pandith, A.H. Removal of heavy metal ions from aqueous system by ion-exchange and biosorption methods. *Environ. Chem. Lett.* **2019**, *17*, 729-754.
